# Reasons for COVID-19 vaccine refusal among people incarcerated in Canadian federal prisons

**DOI:** 10.1371/journal.pone.0264145

**Published:** 2022-03-09

**Authors:** David Ortiz-Paredes, Olivia Varsaneux, James Worthington, Hyejin Park, Shannon E. MacDonald, Nicole E. Basta, Bertrand Lebouché, Joseph Cox, Shainoor J. Ismail, Nadine Kronfli

**Affiliations:** 1 Centre for Outcomes Research and Evaluation, Research Institute of the McGill University Health Centre, Montreal, Quebec, Canada; 2 Correctional Service Canada (CSC), Ottawa, Ontario, Canada; 3 Faculty of Nursing, University of Alberta, Edmonton, Alberta, Canada; 4 School of Public Health, University of Alberta, Edmonton, Alberta, Canada; 5 Department of Epidemiology, Biostatistics and Occupational Health, School of Population and Global Health, McGill University, Montreal, Quebec, Canada; 6 Department of Family Medicine, Faculty of Medicine and Health Sciences, McGill University, Montreal, Quebec, Canada; 7 Division of Infectious Diseases and Chronic Viral Illness Service, Department of Medicine, McGill University Health Centre, Montreal, Quebec, Canada; 8 Division of Immunization Programs and Pandemic Preparedness, Centre for Immunization and Respiratory Infectious Diseases, Public Health Agency of Canada, Ottawa, Ontario, Canada; 9 Metro City Medical Clinic, Edmonton, Alberta, Canada; University of North Carolina at Chapel Hill, UNITED STATES

## Abstract

**Background:**

Vaccine uptake rates have been historically low in correctional settings. To better understand vaccine hesitancy in these high-risk settings, we explored reasons for COVID-19 vaccine refusal among people in federal prisons.

**Methods:**

Three maximum security all-male federal prisons in British Columbia, Alberta, and Ontario (Canada) were chosen, representing prisons with the highest proportions of COVID-19 vaccine refusal. Using a qualitative descriptive design and purposive sampling, individual semi-structured interviews were conducted with incarcerated people who had previously refused at least one COVID-19 vaccine until data saturation was achieved. An inductive–deductive thematic analysis of audio-recorded interview transcripts was conducted using the Conceptual Model of Vaccine Hesitancy.

**Results:**

Between May 19-July 8, 2021, 14 participants were interviewed (median age: 30 years; n = 7 Indigenous, n = 4 visible minority, n = 3 White). Individual-, interpersonal-, and system-level factors were identified. Three were particularly relevant to the correctional setting: 1) *Risk perception*: participants perceived that they were at lower risk of COVID-19 due to restricted visits and interactions; 2) *Health care services in prison*: participants reported feeling “punished” and stigmatized due to strict COVID-19 restrictions, and failed to identify personal benefits of vaccination due to the lack of incentives; 3) *Universal distrust*: participants expressed distrust in prison employees, including health care providers.

**Interpretation:**

Reasons for vaccine refusal among people in prison are multifaceted. Educational interventions could seek to address COVID-19 risk misconceptions in prison settings. However, impact may be limited if trust is not fostered and if incentives are not considered in vaccine promotion.

## Introduction

Canadian correctional settings have witnessed several large SARS-CoV-2 outbreaks since the start of the pandemic [1]. This is likely due to close living conditions, overcrowding, an aging and comorbid population, and the challenges in accessing and implementing effective infection prevention measures [2, 3]. Consequently, the Canadian National Advisory Committee on Immunization (NACI) prioritized “congregate” settings such as correctional settings for early COVID-19 vaccination [4]. However, vaccine uptake rates have remained historically low in Canadian prisons despite the availability and promotion of routine vaccination since the 1990s [5]. This might be due to the disproportionate incarceration of people experiencing social and health inequities and the overrepresentation of hard-to-reach populations, who tend to be less engaged in health promotion and prevention programs [5]. Maximizing COVID-19 vaccine acceptance is therefore essential in preventing individual-level morbidity and mortality among the 30,000 adults currently incarcerated in Canadian federal and provincial/territorial prison each day [6–10].

People incarcerated in Canadian federal prisons were offered the Moderna COVID-19 vaccine, shortly after their prioritization by NACI [11]. The COVID-19 vaccine, like all other vaccines offered in federal prisons, is not mandatory. Despite several mass vaccination campaigns across all 43 federal sites, vaccination uptake remains low at some sites [11]. Understanding reasons for COVID-19 vaccine refusal is key to optimizing vaccine acceptance, achieving herd immunity, and crucial in preventing and mitigating future outbreaks and the consequent harms in correctional settings.

Very few studies have thus far sought to understand reasons for vaccine refusal among people in prison [5, 12, 13]. Given the unique environment, reasons for vaccine refusal might differ from those in the general population; for instance, distrust is lived differently in environments with a history of unethical research and experimentation [14]. This precludes the generalizability of population-based studies to people in prison. Several proposed models of vaccine refusal highlight that attitudes towards vaccination are the result of complex interactions between different social, cultural, and personal factors in vaccine decision-making [15–19]. To increase vaccine confidence, and subsequently, uptake in prison, studies are needed to understand which *modifiable* factors exist for people in correctional settings so that focused interventions can be designed and disseminated. In turn, such interventions may have a positive influence on surrounding communities when incarcerated individuals are released. Given these knowledge gaps, we aimed to better understand reasons for vaccine refusal among people in Canadian federal prison settings.

## Materials and methods

### Study design and participants

We conducted a qualitative descriptive study [20, 21] in three Canadian federal correctional facilities, where adult individuals serve sentences of two years or greater [22]. In collaboration with Correctional Service Canada (CSC), three maximum security (single cell occupancy) all-male federal prisons were chosen as the study sites. They represented the prisons with the highest proportion of COVID-19 vaccine refusal at study inception. Participants were recruited from Kent Institution (KI; British Columbia), Edmonton Institution (EI; Alberta), and Millhaven Institution (MI; Ontario). At the time of the study, 36%, 45%, and 46% of incarcerated individuals at KI, EI, and MI had refused COVID-19 vaccination, respectively. KI houses 253 men; over one-third (92; 36%) are Indigenous and one-quarter (65; 26%) are from ethno-racial and visible minority groups (e.g., Asian, Black, Hispanic). EI houses 267 individuals; more than half (156; 58%) are Indigenous and a minority are from other minority groups (34; 13%). MI houses 248 individuals; over one-quarter are Indigenous (70; 28%) and one-third are from visible minority groups (86; 35%). None of the sites had experienced a widespread COVID-19 outbreak prior or at the time of the study [1].

To participate, individuals had to have previously declined COVID-19 vaccination and provide verbal or written consent in English or French. Individuals who posed a security risk to the research team were excluded. Participants were selected using purposive sampling, a non-probability, flexible sampling strategy in which researchers rely on their judgement to identify available information-rich individuals based on criteria of interest [23]. Given anticipated recruitment challenges due to the security level of the selected sites, we aimed for maximum variation sampling with a focus on ethno-racial identity as the criterion of interest. More specifically, we selected participants from among three ethno-racial categories in accordance with Statistics Canada Standards [24]: White, Indigenous (i.e., First Nations, Inuit, Métis), and visible minority (i.e., those who self-identified as neither Indigenous nor White). No relationship was established with participants prior to study commencement. As per CSC regulations, participants did not receive an honorarium for their participation. This study was approved by the McGill University Health Centre Research Ethics Board (REB # 2022–7868). Once the study background and purpose was explained, participants consented to the use of their demographic information and anonymized quotes for dissemination in scientific journals.

### Data collection

DOP (male Colombian general physician with master’s training in qualitative methodologies) and NK (female scientist, public health researcher, and infectious disease clinician with particular interest in developing evidence-based health care models for people in prison) conducted individual semi-structured interviews in-person, via telephone or via an online videoconferencing software. Interviews were approximately one hour in duration. The interview guide focused primarily on exploring reasons for vaccine refusal (see S1 Appendix). When possible (n = 8), a note taker observed the interview. Thereafter, the interviewer and observer discussed the main content of the interview to complement field notes. All interviews were audio-recorded and transcribed verbatim. Transcripts were not reviewed by participants. Interviews were conducted until data saturation was achieved–defined as the degree to which new interview data tends to repeat or be redundant to that collected in previous interviews [25].

### Theoretical framework

This study was informed by the Conceptual Model of Vaccine Hesitancy [CMVH; 15]. The CMVH considers attitudes towards vaccination as complex, multifaceted, individual decision-making processes affected by emotional, cultural, social, spiritual, political, and cognitive influences. Specifically, in addition to individual factors (i.e., knowledge, past experiences, perceived importance of vaccination for health, risk perceptions, religious/moral convictions, trust), this framework considers attitudes towards vaccination to be influenced by relationships with other individuals such as health professionals, family, and friends. The CMVH also considers the time and context in which vaccination occurs, as well as the role of public health, vaccine policies, and the media in shaping attitudes towards vaccination. Therefore, using the CMVH lens, we considered the balance of attitudes towards vaccination as a continuum from vaccine acceptance to vaccine refusal.

### Data analysis

Interview transcripts and field notes underwent an inductive–deductive thematic analysis. Initially, data was inductively analyzed following the six-step thematic analysis approach described by Braun and Clarke [26]. This allowed the identification of patterns and recurrent ideas across transcripts. Finally, resultant themes were deductively categorized using the vaccine hesitancy factors of the CMVH. DOP conducted a preliminary analysis with NVivo 12® for Mac. The analysis was then further developed by DOP and NK to rework and identify the themes. Results and interpretations were then discussed with co-authors. Participants did not provide feedback on the findings.

## Results

A total of 12 individuals refused to participate. Data saturation was achieved after interviewing 14 participants (median age: 30 years; range 24–46), including three White, seven Indigenous (n = 5 Métis and n = 2 First Nations), and four from other ethno-racial and visible minority groups (n = 4 Black). These interviews took place between May 19 and July 8, 2021. The median incarceration time among study participants was 6 years (range 0.5–18). A total of six people were interviewed at KI (n = 3 White; n = 2 Indigenous; n = 1 visible minority), five at EI (n = 3 Indigenous; n = 2 visible minority), and three at MI (n = 1 Indigenous; n = 2 visible minority). Of the 14, three reported ever having previously accepted influenza vaccination. No repeat interviews were carried out.

**Fig 1:**
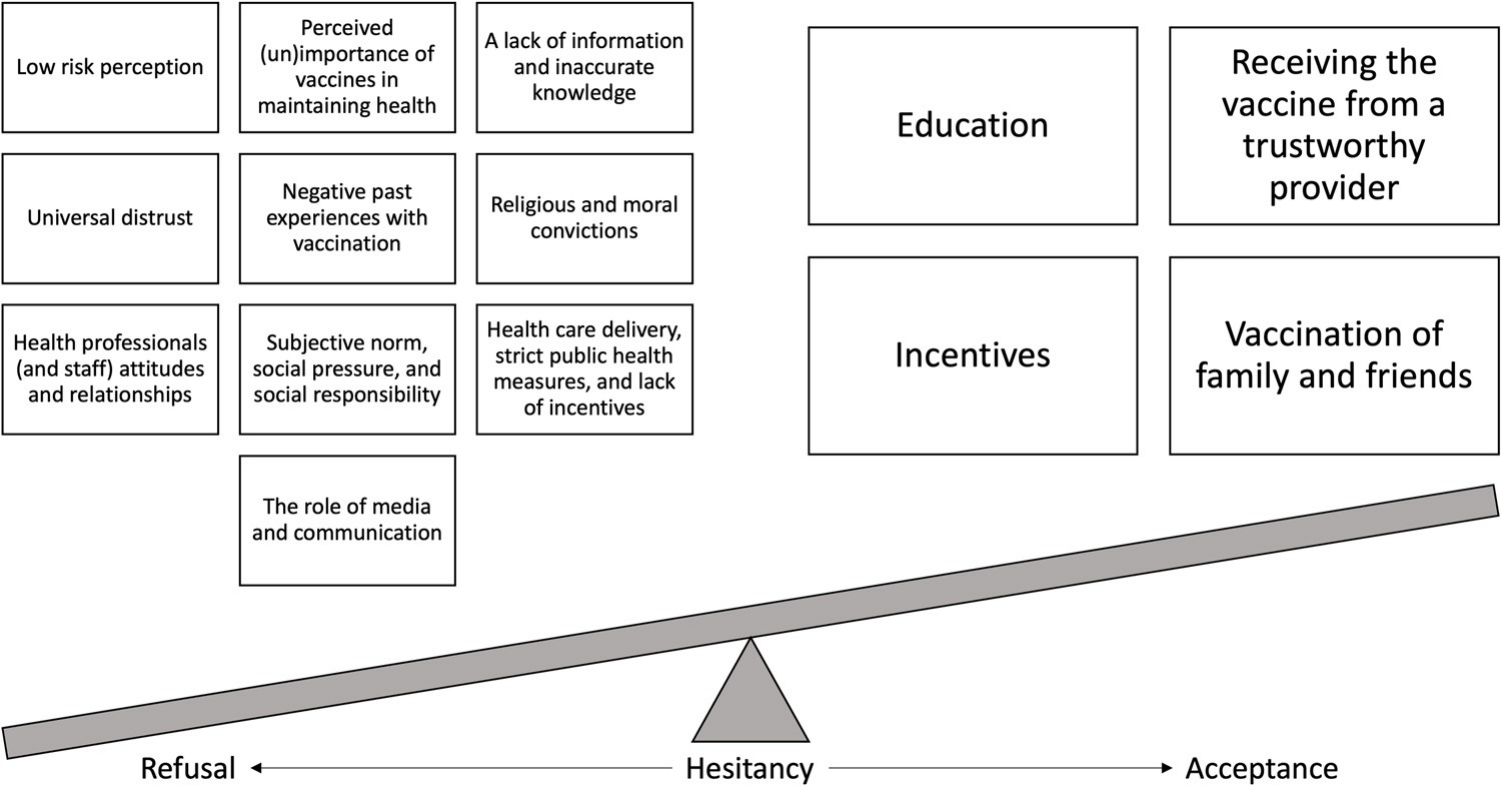
Reasons for COVID-19 vaccine refusal in Canadian federal prison settings and variables that could lead to acceptance.

According to the thematic analysis, all factors within the CMVH were identified in the data. They are organized below according to three levels: individual, interpersonal, and system. Factors that influenced COVID-19 vaccine refusal are outlined in Fig 1.

### Individual-level factors

#### Risk perception

“Proper people think twice” (MI-3)

Participants juggled their perceived risk and severity of COVID-19 with their perceived risks of the vaccine. Some participants described COVID-19 as potentially life-threatening and recognized that incarceration was associated with a higher risk of infection: “It’s hard to physical distance from people (…) I’m sure [COVID-19] would spread around the unit easily, so I would say we’re at a pretty high risk being that our populations are as condensed as they are” (KI-1).

Despite this perception, all participants articulated that they believed themselves to be at lower risk of COVID-19 as a consequence of restricted visits and limited social interactions infection prevention and control measures that were instituted at CSC facilities during the COVID-19 pandemic. They also perceived that being isolated from the community served to protect them, thereby decreasing their risk of acquiring SARS-CoV-2, to the point where COVID-19 vaccination was no longer necessary. The following quotations illustrate low COVID-19 risk perception:

[Getting the COVID-19 vaccine] doesn’t make sense for someone in my position (…) when I don’t interact with anybody in the community, I don’t talk or be around anybody in the community (…) I don’t need it right now (KI-5).

[With respect to the vaccine] I don’t think it’s that important for me being locked up for two years (…) it’s a lot harder to get COVID in here because we’re not able to go out into the world and catch it. We’re locked up on a range with no COVID. (MI-3)

Vaccination willingness was further impacted by participants’ perceptions of the COVID-19 vaccine as unsafe and harmful. Participants expressed a concern that the COVID-19 vaccine was new, developed quickly, and whose mechanism was poorly understood: “They had to do the vaccines fast, right? Like, they had to do it really, really, really fast, just to produce it to the population without even actually figuring what the vaccine is all about” (EI-5). In addition, most participants mentioned feeling worried about possible side effects, particularly long-term. They feared Alzheimer’s disease, strokes, cancer, infertility, and even death secondary to the vaccine. These beliefs led participants to question the need for the COVID-19 vaccine:

Cause [we are] still learning about it because it’s still fresh and a new vaccine (…) We don’t know what the vaccines are going to do in two years, five years, 10 years, we don’t know what they’re going to do (…) So like let me see where this roller coaster is going (KI-4).

People are taking a shot and then they’re getting like a blood infection or what not. And then they’re dying from it. (…) There’s been like lots of cases where people have died and that’s just facts. So it’s like, the potential of the dying from taking a shot (MI-3).

In summary, participants were less interested in COVID-19 vaccination secondary to their low-risk perception of COVID-19 during incarceration in addition to the possible risks associated with the vaccine. Participants mentioned that their risk assessment—and therefore willingness to receive the COVID-19 vaccine—could change if they were in a situation with increased social interactions (e.g., being employed during incarceration, release into the community) or if their prison was the site of a SARS-CoV-2 outbreak.

#### Perceived (un)Importance of vaccination in maintaining health

“Because I don’t have a broken leg, I don’t need a crutch” (KI-3)

Participants perceived their immune systems as strong and robust, and that COVID-19 vaccination was only necessary for those considered weak and vulnerable. Some participants mentioned rarely becoming sick and, given that they had never acquired SARS-CoV-2 despite being possibly exposed, as evidence that they were healthy enough not to need vaccination:

I have, a strong immune system, (…) I don’t get a cold very often. Maybe I’ll get one cold a year (…) my immune system is strong. So why would I go and take a vaccine? (KI-3).

I have strong genes, right? I was around people that got COVID (…) we’re all smoking joints together. And then, apparently, there were some cases there and I never caught it (EI-5).

Participants also referred to good personal hygiene and the incorporation of infection prevention and control measures (e.g., wearing masks) into their habits as strategies that would not only prevent COVID-19 but also preclude the need for vaccination: “It’s like you *might* die or that you *might* get really sick, it’s a *might*; okay, I’ll wash my hands extra four times a day, cover my mouth when I cough and sneeze, wear a mask; just be clean” (KI-4). In addition, participants expressed a preference to maintain health through “natural” immunity rather than vaccination: “I’m one of the people that would rather get sick and build the immunities naturally, than stick it in my body. (…) I don’t like things entering my body that aren’t naturally supposed to be there” (KI-6).

Participants thus refused vaccination due to both the perception that their immune systems were strong and a desire for “natural immunity”, underscoring that vaccination is unnecessary in maintaining health.

#### A lack of information and inaccurate knowledge

“I’m not gonna put stuff into me that I don’t know what the hell it is” (EI-3)

Insufficient information and inaccurate knowledge vis-à-vis the COVID-19 vaccine were important reasons for vaccine refusal. Participants expressed desiring more vaccine information prior to accepting it: “I don’t understand the vaccine. (…) I value my life and if I don’t know the certain information that I want to know, I’m not going to take it” (MI-3). Participants expressed believing they were ill-informed: “I don’t know anything about the COVID-19 vaccine. I know very little (…). There was nothing that educated us” (KI-3). They expressed interest in knowing how the COVID-19 vaccine was developed and tested, its components and mechanism of action, effectiveness, and potential side effects.

This reported lack of information was also found to contribute to conspiracy theories, further dissuading individuals to get vaccinated. One participant (KI-5) described that having inadequate information and unanswered questions could foster conspiracy theories, which could further drive vaccine refusal: “I feel [conspiracy theories] come from people that need a solution to a question they want answered (…) I think deep down it comes from yourself wanting to believe something that’s not true and looking for something to prove it.” Participants expressed that COVID-19 was a “plandemic” (KI-2), created to “destroy the weak” (EI-4), and achieve population control. These misconceptions had a negative impact on willingness to be vaccinated.

#### Universal distrust

“It’s a lack of trust of everything” (EI-4)

A universal distrust, not limited to the correctional system, contributed to vaccine refusal among participants. Participants expressed distrust in the Canadian government and in health authorities, whom they identified as responsible for vaccine development and distribution. This feeling of distrust was amplified by historical trauma and a negative perception on how governments have treated incarcerated individuals and visible minorities in the past:

The Tuskegee Project. When they took a bunch of black people and they gave them all syphilis, and then made it look like they were helping them (…) especially black families, we don’t trust all this stuff. We don’t trust no vaccine (…) Our oppressor wants to come and give us those vaccines, and we’re expected to trust that? (MI-1)

Distrust in the government often fostered scepticism about COVID-19 vaccine development and effectiveness: “I don’t trust the government either. I’d rather not take this COVID shot. And everybody’s dying, it’s because the government made this. I’d rather not in 10 years just die” (EI-4).

In addition to a distrusting the government, participants also expressed distrust in the correctional system. This lack of trust involved all prison employees, including health care providers. Participants expressed feeling mistreated and the objects of experimentation:

It’s not the fact that it’s good or not good, it’s the fact that there’s not trust around it in the first place (MI-1).

I’m very leery about vaccines because over the years I’ve been in a jail, there was a couple of times where I felt like I was the guinea pig for vaccines. This is why I never took them. For the COVID [vaccine] the same thing (KI-3).

Thus, while universal distrust prevented participants from agreeing to vaccination, some participants expressed an openness to vaccination following release or at a location not affiliated with prison, underscoring the degree of distrust in the correctional system. One participant said: “What would influence me [to get the COVID-19 vaccine]? Bring me to a hospital (…) I would trust a nurse at a hospital rather than somebody in this jail” (KI-2).

#### Negative past experiences with vaccination

“It’s a little scary for me to see another inmate sick for a whole day” (KI-3)

Previous negative experiences with vaccines shaped participants’ current attitudes towards vaccines in general and to the COVID-19 vaccine in particular. One’s own negative experiences included fear of needles and previous vaccine-related side effects such as fever, vomiting, and flu-like symptoms. Similarly, some participants witnessed their incarcerated peers experience side effects following COVID-19 vaccination that contributed to their own refusal:

The one time in my life that I took the flu shot, I got so sick that it traumatized me (…) I would feel overheated, I felt like I was burning up inside. So bad that it traumatized me when I was younger. So ever since then, I never touched a vaccine ever again (MI-2).

So two of my friends in here that got vaccinated were put down for the next three days with extreme headaches lying in bed, not feeling well (…) their body just had a hard time processing. I don’t know which one they took. I didn’t look into it that far. I just know it’s not for me (KI-6).

#### Religious and moral convictions

“I don’t have to worry. I believe in the Great Spirit” (EI-5)

COVID-19 vaccine refusal was also influenced by participants’ religious and moral beliefs regarding health and immunity. Some stressed the importance of faith in remaining healthy and defending oneself from COVID-19: “I’m an Indigenous person. We believe that COVID-19 is a spirit (…) we can keep it away by smudging and praying (…) the reason why I don’t have COVID is because, I’m engulfed in my spirituality, and I smudge” (KI-3).

Other reasons around vaccine refusal centred around strict religious convictions. For example, some expressed vaccines as “not permitted” (KI-4) in their faith, while others believed that vaccination could interfere with the natural course of humanity. One participant said: “If I’m looking at it religiously, (…) you’re playing God, right? Because that takes away [from] natural selection. (…) When you give these vaccines, you guys are fighting natural selection and you’re changing the course of the world” (MI-1).

### Interpersonal-level factors

#### Subjective norm, social pressure, and social responsibility

“There’s a lot of doubt that gets put in my head from my visitors” (KI-1)

Participants’ views towards vaccination were often influenced by their support systems, underscoring how subjective norms and social pressures played a role in participants’ decisions to refuse COVID-19 vaccination. Family and friends who refused vaccination, advised against it, or questioned its benefits were examples of the role of a support system in dissuading vaccination: “Just everyone, everyone’s not getting it. I’m pretty sure my whole family, my mom, my brothers and sisters, nephews, nieces, my kids. Nobody’s getting the vaccine” (EI-3). Some participants described feeling greater trust in their support systems than in the recommendations made by health care providers: “They’re closer to me. I would give more consideration to my visitors opinions and what their justifications would be than the health professional here” (KI-1).

From the standpoint of social responsibility, participants expressed not needing to contribute to herd immunity as a reason for vaccine refusal. The idea that 100% vaccination is not required to achieve population-level immunity led some to believe that others could do their part: “Others will take [the COVID-19 vaccine]. It doesn’t have to be me. Eventually we’ll go back to normal once there is a herd immunity. Everyone doesn’t have to be vaccinated, a proportion will be enough” (EI-1). Others mentioned that since the majority of those incarcerated in their range were vaccinated, their refusal would not impact immunity:

More than 70% that are taking the vaccine (…) I think it would be unnecessary or unneeded for, for me to take the vaccine. (…) Seeing more people get vaccinated for me personally makes me feel less and less required to take it (…) I just feel like the onus is coming off (KI-5).

Interestingly, some experienced pressure internally from other incarcerated individuals. A few participants felt pressured to accept the vaccine because others believed that restrictions could be lifted if most individuals in prison were vaccinated: “[They] think that once we’re fully vaccinated, we’ll get visits or we’ll get to do stuff, but there’s nothing on paper saying that. (…) I’m not going to do something because somebody thinks that it’s going to benefit us” (KI-6).

Thus, participants’ relationships with those around them, including peers, friends, and family influenced their attitudes towards the COVID-19 vaccine. While these relationships contributed to vaccine refusal, they could simultaneously encourage vaccination. Some participants were open to receiving the COVID-19 vaccine if they witnessed its receipt by family and friends: “Maybe I’d be like, I don’t want to be the only one in my family not to get it. Maybe if everyone else got it (…) then I would [get the COVID-19 vaccine]” (MI-3).

#### Health professionals (and staff) attitudes and relationships

“When you were in need, they walked away from you. But now they wanna give you a COVID shot?” (MI-1)

Participants’ decisions to refuse the COVID-19 vaccine were also shaped by their relationships with health care professionals and prison staff. Indeed, reported stigmatizing attitudes from, and negative experiences with, prison staff (including health care providers) decreased their willingness to receive the vaccine. On occasion, individuals’ negative experiences were the result of miscommunication with health care providers or feeling that they had not received adequate information following brief interactions:

Doctors, I’d say they’re absolutely disrespectful. They’re biased. They’re ignorant. (…) They don’t even listen. (…) I’ve had many questions about [the COVID-19 vaccine] and they [say]: ‘oh, we’ll talk later, we don’t have time.’ It’s been six months I’ve been asking about this vaccine. They haven’t answered one of my questions. (…) We should be able to trust this vaccine. It’s just right down to another lack of communication (KI-2)

Prison employees that failed to follow infection and prevention measures and that refused the COVID-19 vaccine represented a threat to participants’ perceived sense of protection (from isolation) and were viewed as the most likely sources of infection. Some participants thus suggested that vaccination efforts should be directed towards prison staff instead.

A lot of staff don’t even wanna take the COVID vaccine (…) if the COs [correctional officers] were following lockdown procedures, we wouldn’t be getting COVID. If the COs were wearing their masks properly, we won’t be getting COVID. If the COs weren’t spreading it to each other, we wouldn’t get COVID (MI-1)

Thus, prison staff attitudes and their relationships with incarcerated individuals had an impact on how participants perceived their risk of COVID-19, and amplified feelings of distrust with prison personnel, including health providers, ultimately leading to COVID-19 vaccine refusal.

### System-level factors

#### Health care services in prison: Health care delivery, strict measures, and a lack of incentives

“We’re being criminalized, we’re being demonized (…) There’s no doors that are opening for us. There’s no incentives, there’s only harm” (MI-1)

The health care services provided in prison, in addition to the infection prevention and control measures that were instituted, seemed to have a negative impact on participants’ willingness to receive the COVID-19 vaccine. Perceived pre-existing delays in health care services influenced COVID-19 vaccine acceptance. Some participants felt frustrated that other health services they felt were more important (e.g., mental health) were not delivered efficiently in comparison to COVID-19 vaccine rollout and delivery:

I’ve been looking for medical help for so long, so long, but then the medical comes quickly to give me a COVID vaccine (…) I’m like, ‘I’ve been looking for this, for this long, but you guys so quickly can do this for me?’ (…) If they showed the same care for everything like they do in COVID, I would be like, ‘Yeah, no problem’ (MI-1).

Participants also felt stigmatized and punished secondary to COVID-19 restrictions. They perceived that infection prevention and control measures such as mandatory quarantine, physical distancing, and the prohibition of inter-range interactions were strict and were introduced to seek greater control:

You’re only supposed to be there for 14 days; I was there for 28 days (…) If a guy came by my cell and stopped and talked to me, they’d say, ‘Oh, you were in contact with somebody that had COVID,’ then I’d get another 14 days stuck there. Stupid shit. We’re only coming out of our cells for 20 minutes, 10 minutes a shift, the morning/afternoon shift (EI-3).

Why would we be separated? It literally makes no sense. It’s just something to manage us, or either they want more control, or they want to make our lives fucking shittier (EI-4).

As a result of this negative perception and the frustration generated by the restrictions, some participants felt that SARS-COV-2 was fictitious and mild: “when you have to go through [restrictions] like that (…) COVID-19 starts to become, like a joke (…) people start to believe, like it’s not really important” (MI-1).

Finally, a lack of incentives, both material and non-material at the time of vaccination and thereafter, influenced COVID-19 vaccination refusal. Participants failed to identify personal benefits in getting vaccinated in the absence of incentives:

I lost everything, and now society wants me to get a shot to help [keep] them from not getting sick. Well, no, no. How about that? What do I get out of this? Maybe I want it, maybe I want to get sick and die now. I’m just me. What’s it gonna take? (KI-2)

[The COVID-19 vaccine] doesn’t benefit me as an inmate in any way. (…) Even if I’m fully vaccinated, we’re still not getting our visits back. We still have to wear masks everywhere. There’s no benefit for being vaccinated for an inmate. (KI-6)

Participants mentioned that material incentives such as money, phone cards, and food could motivate them to consider vaccination: “if you get the full vaccine, (…) it’s like the little things that people want in here. (…) Canteen free for three months, (…) have pop and a chocolate bar or something like that” (MI-3). In addition, participants did not feel incentivized to accept the COVID-19 vaccine as they failed to witness non-material incentives in the form of changes in COVID-19 restrictions following vaccination. Participants proposed the lifting of mandatory quarantine for vaccinated individuals, in-person visits, inter-range interactions, access to the gym, and television time as ways to increase vaccination: “If you tell people in-person visits [incentives], (…) there would be a lineup (…) because people just want to see their family (…) and if it’s a vaccine that will let them do that, count everybody into the vaccine” (MI-1).

A minority expressed that incentives would not impact COVID-19 vaccine acceptance. Some even recognized the tensions between the ethical concerns of incentives versus their potential benefits:

I feel like if I was pressured into not getting my visits back until I get this vaccine, I’d feel like that’s sort of against my rights (…) because visits [are] one of the rights that we have here. It’s not a privilege, (…) but I think incentives would be the only way (KI-5).

#### The role of media and communication

“I’m stuck in my cell. So it’s like, I’m just watching the TV all the time” (MI-3)

Television is the only source of media that participants can access while incarcerated. Information spread by mainstream media or accessed prior to incarceration also influenced participants’ decisions to refuse COVID-19 vaccination. Participants expressed learning about vaccine side effects including thromboembolic events (i.e., blood clots) and breakthrough cases among fully vaccinated individuals through the media. This content influenced participants’ decisions by changing their risk perception of the vaccine: “Maybe not seeing that stuff on the news, that woman catching blood clots in the brain. You guys should have just kept that to yourselves and then, I would have got it [the COVID-19 vaccine]” (EI-2).

Participants often expressed feeling apathetic towards the COVID-19 pandemic and the vaccine as a result of the media. They felt that the news was constantly discussing the pandemic, either locally or globally, which contributed to feelings of disinterest and to vaccine refusal: “You’re desensitizing us (…) Do I have to hear about COVID 24/7? We know we’re in a pandemic. We know all this stuff. We don’t need to hear it every single day” (MI-1).

Others perceived COVID-19 media content as inaccurate, with some expressing a fear that it was “brainwashing people” (EI-5). Similarly, participants felt COVID-19 was exaggerated: “It seemed to be a lot of media hype (…) So it seems like it’s much more trumped up then the actual virus is capable of” (KI-1).

In summary, media content had an important role in shaping participants’ views of the COVID-19 vaccine. Participants seemed to trust the content that reinforced vaccine scepticism (e.g., news on side effects) and questioned information aimed at promoting vaccine uptake (e.g., news on the severity of COVID-19).

## Discussion

This qualitative descriptive study explored reasons for COVID-19 vaccine refusal among people incarcerated in three Canadian federal correctional institutions representing prisons with the highest proportion of COVID-19 vaccine refusal at study inception. We found that COVID-19 vaccine refusal was impacted by multiple factors across several dimensions—individual, interpersonal, and system–each of which potentially influenced one another and led to vaccine refusal. According to the CMVH, the theoretical framework used in our study, vaccine hesitancy and refusal are decision-making processes whose determinants are not only complex but are also *context-specific* [13, 15]. Consequently, we identified three factors that were particularly relevant to the correctional setting: risk perception, lack of incentives in prison-based health care services, and distrust—all of which are modifiable and could serve as the focus of future interventions.

The three context-specific factors that we identified in our analysis are potentially modifiable, suggesting that it may be possible to decrease vaccine refusal among people in prison. We found that participants perceived their risk of COVID-19 to be low due to restricted visits and interactions—a situation that is likely unique to SARS-CoV-2 given the exceptional infection prevention and control measures that were instituted in prisons [5, 12]. Similarly, we found that participants reported being misinformed about vaccine-related side effects secondary to a lack of information, which negatively influenced their perception of the COVID-19 vaccine. Both facets of risk perception (i.e., risk of disease and risks of vaccination) could be simultaneously addressed through education. Second, participants failed to identify personal benefits of vaccination due to the lack of incentives. While incentives are not routinely offered during vaccination in Canadian federal and provincial prisons, the promotion of health care through incentives could be considered to decrease SARS-CoV-2 related cases, hospitalizations, and deaths and accelerate a “return to normal” in correctional settings. Finally, participants refused COVID-19 vaccination in part due to the negative relationships that exist with prison-based health care providers, resulting in a high level of distrust. Thus, in addition to education and incentives, building trust with those incarcerated will be critical moving forward [27].

As misinformation was identified as an individual-level reason for vaccine refusal, education will likely be an essential component in improving COVID-19 vaccine uptake in correctional settings. All participants reported existing COVID-19 vaccine information in prison as minimal, incomplete, or inadequately tailored to the needs of incarcerated people. Participants identified a need for additional information vis-à-vis COVID-19 vaccine development and testing, its components and mechanism of action, effectiveness, and potential side effects—concerns also identified by people in the community [28]. Educational interventions could thus not only seek to address individuals’ low COVID-19 risk perception—prisons remain high-risk settings despite preventative measures as evidenced by outbreaks—but could also seek to improve knowledge about the COVID-19 vaccine. Furthermore, educational material could attempt to address incarcerated peoples’ false perception of COVID-19 vaccine futility as a result of the “lay theory of immunity” [15]. While the content is important, reflecting on those best positioned to deliver the educational material is primordial as well. For example, peer-led education sessions may enhance their impact [29, 30]. Educational interventions may also have a positive effect beyond prison settings. As more knowledgeable individuals are released into community, they may spearhead changes in their support systems. While education is inarguably important, studies have shown that educational interventions only modestly increase vaccine uptake in prison settings [31–33]. Therefore, novel educational approaches such as the adoption of a family-centred health promotion perspective [34], that is, expanding the provision of information to incarcerated individuals’ support network, could be considered. This approach would have the additional benefit of addressing subjective norm—a reason for COVID-19 vaccine refusal identified in our study. Despite the importance of educational interventions, education alone is unlikely to be sufficient in improving COVID-19 vaccine uptake in prison. Education will thus likely need to be paired with other strategies, including incentives.

Incentives could be considered for future COVID-19 vaccination campaigns in prison settings. Incentives have been shown to be effective strategies in health promotion in changing human behaviours [35–38]. Financial incentives have been historically used in prison settings to promote adherence to tuberculosis treatment [39]. While incentives (e.g., financial honoraria) are considered standard of practice for research study participation, some have argued that the use of incentives—either financial or non-financial—with people in prison, even in the context of research, may be considered unethical and/or coercive, particularly in the context of historical correctional trauma [40]. Indeed, prison-based incentives may fuel distrust and sentiments of inequity and injustice among certain individuals, thereby having an opposite desired effect [37, 41, 42]. Conversely, others have argued that it is discriminatory and unethical to deny incentives to offenders, particularly in the context of research participation [43]. In fact, some United States state prisons have offered monetary incentives to increase COVID-19 vaccination acceptance among offenders [44–46]. Study participants identified interest in incentives at the time of vaccination through money, phone cards, and even food, as potential facilitators to COVID-19 vaccine acceptance. Participants also recognized the importance of identifying non-material incentives in the form of “privileges” for vaccinated individuals (e.g., lifting of mandatory quarantine, in-person visits, inter-range interactions, access to the gym). CSC does not currently allow the use of incentives in health service promotion or as compensation for study participation in any of its 43 federal prisons [47]. This is in contrast to some Canadian provincial prisons [47]. While the implementation of incentives in federal prisons as a tool in health promotion would set a new precedent, this approach should first be explored and considered in light of the existing evidence as an opportunity to promote equity and justice among a marginalized population. Indeed, more research is needed to understand both the advantages and potential detrimental effects that incentives for health promotion may have in prison settings. The choice of incentives and “earned privileges” [42] could first be identified using a participatory engagement approach with stakeholders (e.g., correctional and prison leaders, health care providers, prison advocates), ensuring that all stakeholders have the opportunity to have their views and concerns heard.

Fostering trust will be another essential component of improving COVID-19 vaccine uptake among people in federal prisons [27]. Some participants expressed feeling distrust towards all correctional employees, including health care providers. While there are several challenges to building trusting relationships between correctional employees and people in prison, health care providers’ documented struggles with their ‘dual loyalty’ (i.e., to the patient and to the prison administration) is one that cannot be underestimated [48]. Bill C-83, which promotes “professional autonomy and clinical independence” of health care professionals affiliated with CSC, is an important first step towards patient-cantered care and trust-building in prison settings [49]. Nonetheless, to further foster provider-patient trust, studies have shown that health care providers that adopt a dialogue-based approach with vaccine-hesitant individuals, or vaccination campaigns that involve leaders and peers [28, 50] have higher vaccine uptake. In federal corrections, these individuals could be Indigenous Elders or prison committee members. Other studies could also explore the impact of health care professionals not affiliated with the correctional system on vaccine uptake as they may be perceived as more trustworthy. Finally, structural changes and continuing professional development are needed to foster culturally safe prisons environments [51] and to support patient-centred approaches [52]. This may involve the implementation of “inmate volunteer” models of care where incarcerated individuals take more active roles in prison health care delivery. These models have had positive effects on trust-building and the empowerment of incarcerated individuals [53].

There are limitations to our study. Firstly, participant recruitment was difficult both due to the logistical challenges of study participation in maximum security prisons and the ongoing COVID-19 pandemic. While only a limited number of participants were included, they were ethno-racially-representative of the incarcerated population who refused COVID-19 vaccination at the study sites, thereby allowing us to capture diverse perspectives and achieve data triangulation and saturation. Secondly, as with all qualitative research, social desirability and volunteer sampling biases may have been introduced. To limit these biases and to ensure honest dialogue, participants were reminded that they could withdraw from the study without repercussions. That said, similar reasons for vaccine hesitancy have been reported in the Canadian general population, suggesting that social desirability bias was minimal [54, 55]. Despite these limitations, this study is an important contribution to the scarce literature on COVID-19 vaccine refusal among incarcerated individuals [56] and may be generalizable to other correctional settings outside Canada and to populations who may share similar sociodemographic characteristics such as people who inject drugs, homeless communities, and migrants.

## Conclusions

Vaccine refusal among people in prison is complex and is influenced by several individual-, interpersonal- and system-level factors. While some factors will require time and others cannot be modified, our study underscores that several factors could be addressed to increase COVID-19 vaccine uptake. Our study highlights the need for tailored educational interventions for people in prison. However, the impact of these interventions will be limited if trust between incarcerated individuals and health care providers is not fostered and if incentives are not considered in vaccine promotion.

## Supporting information

S1 Appendix Interview guide. (PDF)
