# Supplementary material for: Reasons for COVID-19 vaccine refusal among people incarcerated in Canadian federal prisons
Source: PLoS One. 2022 Mar 9;17(3):e0264145. doi: 10.1371/journal.pone.0264145 (PMC8906611; doi:10.1371/journal.pone.0264145)
Supplement: S1 Appendix — (PDF) [file pone.0264145.s001.pdf]

Supplemental Material

S1. Interview Guide

Background:

1. How old are you?
2. What is your ethnocultural background?
3. What is your highest level of education?
4. How long have you been incarcerated?

Health service(s) received

5. How would you evaluate the care that you receive from the health care worker(s) in prison?
6. To what extent do you typically follow health care workers' recommendations with respect to your health? What would make you follow their recommendations? What would make you not follow their recommendations?

Current COVID experience

7. What has been your experience with COVID-19 so far?
    a. Were there people who had COVID-19 during your time in prison? Other inmates? Staff or health care workers?
    b. Have you or anyone you know had COVID-19?
8. To what extent do you think prisons are at low, average, or high risk for COVID-19 outbreaks? Why? To what extent do you think you are at low, average, or high risk of getting COVID-19? Why?

COVID-19 knowledge sources

9. What do you know about COVID-19? What information have you received about COVID-19 in prison?
10. What else would you like to know about COVID-19 (restricted to disease itself)?

<u>Perceptions of vaccines in general and the COVID-19 vaccine in particular</u>

11. What do you think about vaccines?
    a. How acceptable are vaccines in general for you?
    b. Tell me about your experiences with vaccines both outside and inside the prison.
12. What a vaccine is? How does it work? What does it do to the body?
13. Explain your reasons for declining the COVID-19 vaccine.
14. What are your concerns or fears about the COVID-19 vaccine?
15. What do you know about the COVID-19 vaccine? What information have you received about the COVID-19 vaccine? From whom? In what format? Did you find it useful?
16. What are the benefits in getting the COVID-19 vaccine?
    a. How effective do you think the vaccine offered to you is against COVID-19?
    b. How important is it for people in prison (staff, inmates…) to receive the COVID-19 vaccine? Why?
17. What may influence you to get the COVID-19 vaccine?
    a. What information about the vaccine may influence your decision to get the vaccine?
    b. To what extent has seeing others being vaccinated influenced your views on the COVID-19 vaccine?
    c. Why do you think other inmates have accepted the vaccine?

18. To what extent has someone (health care professional, another inmate, correctional employee, family member) tried to pressure you to either accept or decline the COVID-19 vaccine?
19. What would you like to know about the COVID-19 vaccine?

Final question

20. We talked about COVID-19, the vaccine, and the information sessions that we would like to implement for inmates. Do you have any final thoughts concerning what we discussed?
